# Predictive factors of axillary pathological complete response in HER2-positive breast cancer patients treated with neoadjuvant chemotherapy

**DOI:** 10.1007/s00330-025-12241-5

**Published:** 2025-12-24

**Authors:** Marta Rodríguez de Trujillo Campo-Cossío, Sara Romero-Martín, Beatriz Rodríguez-Alonso, Pilar Font-Ugalde, José Luis Raya-Povedano, Marina Álvarez-Benito

**Affiliations:** 1 Maimónides Biomedical Research Institute of Córdoba (IMIBIC), Córdoba, Spain; 2 Breast Cancer Unit, Department of Diagnostic Radiology, Reina Sofía University Hospital, Córdoba, Spain; 3 University of Córdoba, Córdoba, Spain; 4 Department of Oncology, Reina Sofía University Hospital, Córdoba, Spain; 5 Rheumatology Department, Reina Sofía University Hospital, Córdoba, Spain

**Keywords:** HER2 receptor, Breast neoplasms, Neoadjuvant therapy, Ki-67 antigen, Treatment outcome

## Abstract

**Objective:**

Axillary lymph node dissection (ALND) is associated with significant morbidity in breast cancer patients. In HER2-positive (HER2+) breast cancer, neoadjuvant chemotherapy (NAC) combined with anti-HER2 therapy can achieve high rates of pathological complete response (pCR), supporting the de-escalation of axillary surgery. Identifying reliable predictors of axillary pCR is essential for safely omitting ALND in selected patients.

**Materials and methods:**

This retrospective cohort study included 60 women with HER2+ breast cancer and axillary involvement treated with NAC followed by surgery between 2015 and 2022. All patients had pre- and post-treatment mammography, ultrasound, and MRI. The variables analyzed included age, Ki-67 index, hormone receptor status, breast and axillary radiological and pathological response (rCR, pCR), number of suspicious lymph nodes, and type of axillary surgery. Univariate and multivariate logistic regression models were performed to identify independent predictors of axillary pCR.

**Results:**

Axillary pCR was achieved in 85% of patients. On univariate analysis, axillary rCR (OR = 5.83; 95% CI: 1.30–26.12; *p* = 0.021) and breast rCR (OR = 6.79; 95% CI: 1.27–36.34; *p* = 0.025) were significantly associated with axillary pCR. Multivariate analysis identified axillary rCR (OR = 17.98; 95% CI: 1.43–225.93; *p* = 0.025) and Ki-67 index (OR = 0.90; 95% CI: 0.82–0.99; *p* = 0.045) as independent predictors of axillary pCR.

**Conclusions:**

Axillary rCR and Ki-67 are independent predictors of axillary pCR in HER2+ breast cancer patients treated with NAC. These findings may help optimize axillary management and support less invasive approaches. Further multicenter studies should validate predictive models and refine imaging criteria for axillary response assessment.

**Key Points:**

***Question****Identifying predictive factors for axillary pCR in HER2+ breast cancer patients undergoing NAC*.

***Findings****Axillary radiological complete response predicted axillary pCR in univariate (p = 0.021) and multivariate analyses (p = 0.025), while Ki-67 was marginally significant in the multivariate model (p = 0.045)*.

***Clinical relevance****Recognizing reliable predictors of axillary pCR may support the omission of ALND in selected HER2+ patients, minimizing surgical morbidity*.

**Graphical Abstract:**

**Figure:**
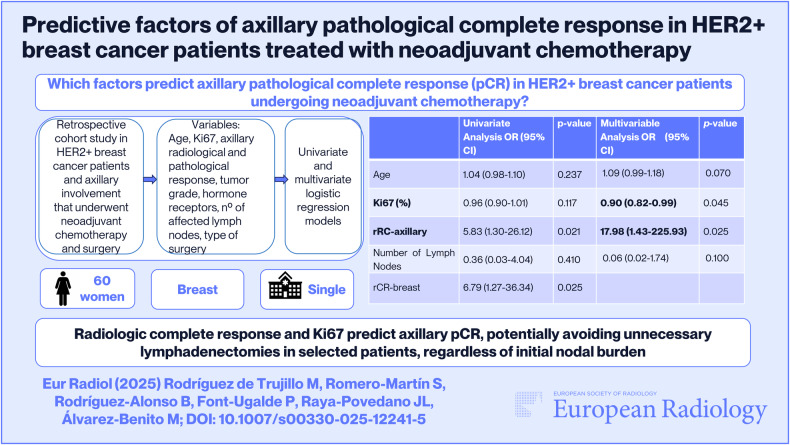

## Introduction

Axillary management in breast cancer has evolved significantly over the years, shifting from radical surgical interventions to more conservative approaches. Traditionally, complete axillary lymph node dissection (ALND) was performed in all women diagnosed with invasive breast cancer to both diagnose and treat axillary disease radically [1]. However, this approach often resulted in significant complications such as lymphedema, chronic pain, and arm mobility limitations [2]. The advent of systemic treatments such as chemotherapy and hormone therapy led to the necessity of less invasive alternatives, leading to the introduction of sentinel lymph node biopsy (SLNB) in the 1990s [3, 4]. This technique allowed for accurate axillary staging with reduced morbidity. Landmark studies, including the National Surgical Adjuvant Breast and Bowel Project B-32 and American College of Surgeons Oncology Group (ACOSOG) Z0011 trials, demonstrated that SLNB was as effective as ALND in patients with negative sentinel nodes or micrometastases [5, 6].

In HER2-positive (HER2+) breast cancer, axillary management has further advanced with the use of neoadjuvant chemotherapy (NAC) combined with anti-HER2 agents. This combination has been shown to induce axillary pathological complete response (pCR) in up to 74% of cases, raising the possibility of avoiding ALND in selected patients [7–11]. The ACOSOG Z1071 study demonstrated that patients with clinically positive nodes who achieve axillary pCR after NAC could safely avoid ALND [12].

Recent guidelines recommend targeted axillary dissection (TAD) for patients with up to three suspicious nodes at initial diagnosis who achieve axillary radiologic complete response (rCR), with the exception of HER2+ cases [13, 14]. In this subgroup, the high rate of axillary pCR observed has led to proposals to expand the criteria from three to four suspicious nodes as an indication to proceed directly to ALND. This approach was analyzed in a study by García-Tejedor, which suggested performing SLNB after NAC in patients with HER2+ or triple-negative breast cancer (TNBC) and a high initial axillary burden (cN2) if a clinical and radiological response is achieved [15]. Similar findings have been reported in international studies, such as those by Caudle et al and Zhang et al, supporting the feasibility of TAD and the omission of axillary surgery in selected cases [14, 16].

In addition to the axillary stage, other factors have been identified as predictors of axillary pCR in HER2+ breast cancer patients, including age, initial tumor size, tumor grade, Ki-67, and axillary radiologic response to NAC [10, 17–26].

In light of these developments, this study aims to analyze the factors that significantly influence achieving axillary pCR in HER2+ breast cancer patients with low (≤ 3 positive nodes) and high (≥ 4 positive nodes) initial axillary burden undergoing NAC, with the goal of reducing unnecessary lymphadenectomies and replacing them with less invasive techniques such as SLNB or TAD, thereby improving patient outcomes.

## Materials and methods

### Study design and patients

This retrospective cohort study was conducted at the Reina Sofía University Hospital in Córdoba, Spain. It included women diagnosed with HER2+ breast cancer and axillary involvement at diagnosis who received NAC followed by surgical resection between October 2015 and November 2022.

**Fig. 1:**
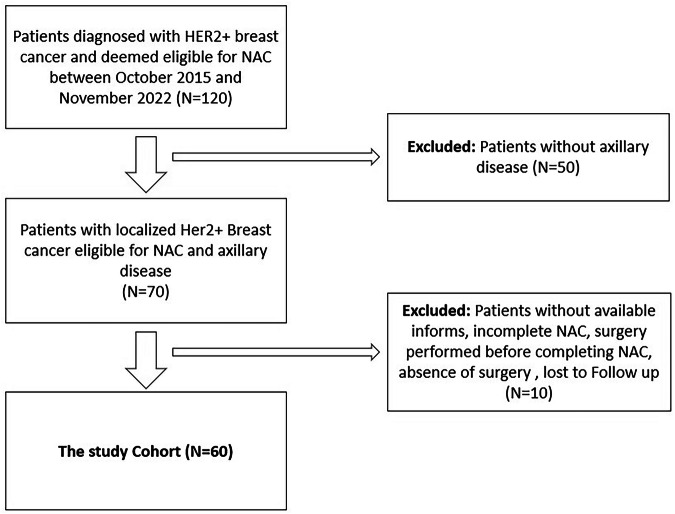
Study population. Flowchart showing patient selection process. The final study cohort included 60 patients who underwent both pre- and post-NAC axillary imaging and subsequent surgical evaluation. Arrows indicate progression through inclusion criteria. NAC, neoadjuvant chemotherapy; MRI, magnetic resonance imaging; pCR, pathological complete response

The inclusion criteria were women aged 18 years or older with histologically confirmed HER2+ breast cancer, axillary involvement verified via biopsy at diagnosis, and completion of neoadjuvant treatment. The exclusion criteria included surgery prior to completing NAC or absence of surgery post-NAC, as well as missing pathology or radiology data (Fig. 1).

The decision to administer NAC was made by the hospital’s Multidisciplinary Breast Committee. All enrolled patients underwent the same imaging study protocol (mammography or tomosynthesis, breast and axillary ultrasound, and breast magnetic resonance imaging) before and after NAC treatment.

## Study protocol

### Imaging technique

Pre-treatment imaging tests were performed at the time of diagnosis, prior to the initiation of NAC and included digital mammography or tomosynthesis with bilateral craniocaudal (CC) and mediolateral oblique (MLO) views, acquired using a single digital mammography device (Lorad Selenia, Hologic) or a single digital breast tomosynthesis device (Selenia 3Dimensions, Hologic); breast and axillary ultrasound with high-resolution linear transducers (7–15 MHz, Canon Aplio i800); and contrast-enhanced magnetic resonance imaging (MRI) (Siemens MAGNETOM Aera 1.5 T and Siemens MAGNETOM Vida 3 T), using axial T2 fast spin echo, Spin echo-echo planar imaging diffusion-weighted imaging (SE-EPI DWI) sequences, and dynamic 3D T1-weighted imaging with one pre-contrast and five post-contrast sequences after intravenous administration of the paramagnetic contrast agent gadoteridol at a dose of 0.10 mmol/kg and a rate of 3 mL/s, followed by a 20 mL saline flush. Post-treatment imaging was conducted two weeks after completion of NAC—approximately 6 months after the initial evaluation—and used the same modalities to evaluate changes in tumor size, lymph node morphology, sonographic features, and dynamic contrast patterns.

### Imaging interpretation

Each patient’s studies were interpreted by one of the hospital’s ten breast radiologists (who had between three and 21 years of experience in breast imaging).

Post-NAC assessment was performed using unilateral digital breast tomosynthesis, ultrasound, and MRI. The breast radiological response was assessed based on the presence or absence of contrast enhancement of the initial lesion on dynamic contrast-enhanced MRI, using adapted Response evaluation criteria in solid tumors criteria and defining complete response to NAC in the breast as the absence of enhancement in the dynamic post-contrast study (breast rCR) [27].

Although axillary lymph node morphology was also evaluated by MRI, final classification of the axillary radiological response was determined using ultrasound findings, taking into account both the number of lymph nodes involved and their morphology. Suspicious lymph nodes were defined according to Bedi’s in vitro classification as type 4 (generalized lobulated hypoechoic cortex), type 5 (focal hypoechoic cortical lobulation), or type 6 (totally hypoechoic node with absent hilum). Axillary rCR to NAC was strictly defined as complete normalization of lymph node appearance by ultrasound, specifically normalization of cortical thickening (< 3 mm) and the restoration of a normal fatty hilum on ultrasound assessment (type 1, 2, or 3 according to Bedi’s in vitro classification), without formal quantitative measurement of the short-axis diameter [28].

## Interventional procedures

The primary breast tumor and any suspicious axillary lymph nodes were biopsied using a 14-G core needle biopsy for histopathological analysis. After the neoadjuvant treatment plan was determined by the multidisciplinary committee, the tumor was marked with a radiopaque metallic clip (HydroMARK^TM^) before starting treatment. Before 2019, suspicious lymph nodes were not marked, and SLNB was performed as the standard axillary procedure after NAC. From 2019 onwards, with the introduction of TAD, at least one suspicious axillary lymph node was marked with a radioactive seed prior to NAC for intraoperative localization using a gamma probe, even in cases of rCR. In accordance with the institution’s protocol, which is based on National Comprehensive Cancer Network Guidelines, up to three nodes (cN1) were marked with seeds to prioritize minimally invasive management of limited nodal involvement [29, 30]. For patients with four or more suspicious nodes (≥ N2), ALND was performed, regardless of imaging response to NAC, as recommended by clinical guidelines during the study period.

## Histopathological evaluation

Pre-treatment biopsy samples were first analyzed by the pathology department to confirm the presence of malignant lesions, determine tumor type, and assess tumor grade following standard diagnostic protocols. Subsequently, immunohistochemistry (IHC) was performed on pre-NAC core biopsy specimens to evaluate the Ki-67 proliferation index as a marker of tumor proliferation, using the MIB-1 antibody according to the recommendations of the International Ki-67 in Breast Cancer Working Group, as well as to assess hormone receptor status (estrogen and progesterone receptors), and HER2 amplification status using fluorescence in situ hybridization (FISH) and/or IHC [31]. Post-NAC, all surgical specimens were examined by a pathologist with expertise in breast oncology. The tumor cellularity reduction was categorized using the Miller-Payne grading system, defining breast pCR as the absence of invasive cancer in the breast (grade 5, 100% cellular reduction) regardless of ductal carcinoma in situ (DCIS) (ypT0/is). Axillary pCR was defined as the absence of residual invasive cancer in lymph nodes (ypN0). This comprehensive evaluation provided critical insights into the treatment response and guided subsequent therapeutic decisions [32].

## Treatment

All patients received NAC with four cycles of doxorubicin plus cyclophosphamide followed by taxanes and dual HER2-targeted therapy (pertuzumab and trastuzumab), according to international guidelines. Surgery was adapted to tumor response and patient preference, including breast-conserving surgery or mastectomy. Axillary management depended on initial nodal involvement and NAC response: from 2015 to 2018, SLNB was performed in patients with ≤ 3 affected nodes and axillary rCR, while from 2019 onward, TAD was implemented in light of new scientific evidence demonstrating greater sensitivity in detecting residual disease [32, 33]. ALND was performed in patients with ≥ 4 involved nodes, lack of radiological response, or residual disease on SLNB/TAD. Finally, adjuvant therapy was individualized and included anti-HER2 therapy, radiotherapy, and/or endocrine therapy based on pathological results.

## Variables and statistical analysis

Data were collected from the patients’ medical records, including radiology and pathology reports. The variables collected included age (years), date of diagnosis, radiological findings (nodule, architectural distortion, calcifications, focal asymmetry, or enhancement on MRI), tumor size (millimeters), histological type, tumor grade (1, 2, or 3), hormonal receptor and HER2 status (positive/negative), Ki-67 proliferation index (percentage, %), number of suspicious lymph nodes (1, 2, 3, 4, or > 4), breast rCR and axillary rCR (yes/no), type of breast surgery (conservative or mastectomy), axillary treatment (SLNB, TAD, or ALND), breast pCR and axillary pCR (yes/no), post-surgical staging (ypT and ypN), and follow-up (months from diagnosis to the occurrence of an oncologic event—recurrence or death—or to the date of the last clinical or imaging review in event-free patients).

Statistical analyses were performed using Statistical Package for the Social Sciences (SPSS) software, version 25. A descriptive study was conducted by calculating the arithmetic mean and standard deviation (SD) for quantitative variables and absolute and relative frequencies (100%) for qualitative variables.

Considering axillary pCR (yes/no) as the dependent variable, univariate logistic regressions were performed to determine the relationship of each variable potentially associated with axillary pCR. The degree of association was estimated using odds ratios (OR) and 95% Cornfield confidence interval (CI). Using the Wald statistic, variables with *p* ≥ 0.15 were individually eliminated from the model (stepwise selection procedure), and a comparison of the reduced model with the model that included the eliminated variables was conducted using the likelihood ratio test. Possible interactions between variables were studied through significant changes in the log-likelihood when the interaction term was introduced. Variables with a significance level greater than 0.05 were analyzed as potential confounding factors and were defined as such if the percentage change in the model variable coefficients exceeded 20%. Cook’s distance was used as a diagnostic test for outlier detection. The Hosmer–Lemeshow statistic was used to assess the goodness of fit. The corresponding ROC (Receiver Operating Characteristic) curve and the area under the curve (AUC) were constructed to determine the predictive capacity of the model. In addition, we estimated test performance measures—sensitivity, specificity, positive predictive value (PPV), and negative predictive value (NPV)—with 95% CIs for axillary rCR (overall and stratified by nodal burden) and for Ki-67 (overall and stratified by < 30% vs ≥ 30%) using the cohort median (30%) as the cut-off value. CIs were calculated using the exact binomial (Clopper–Pearson) method.

All tests were two-sided, and results were considered statistically significant when *p* < 0.05.

## Results

### General results

Between October 2015 and November 2022, a total of 60 women with HER2+ breast cancer and axillary involvement at diagnosis were treated with NAC and subsequent surgery at the Reina Sofía University Hospital. The mean age was 58.93 years (SD 11.73). The mean tumor size at diagnosis was 44.27 mm (SD 26.86), and the mean Ki-67 proliferation index was 37.26% (SD 18.83). The baseline characteristics of the study population are summarized in Table 1.

**Table 1:** Patient demographics, clinical, and histopathology characteristics

	Mean (SD)	N° of patients (%)
Age, years	58.93 (11.73)	
Radiologic finding		
Focal asymmetry		9 (15)
Calcifications		8 (13.3)
Architectural distortion		9 (15)
Nodule		29 (48.3)
MRI enhancement		4 (6.7)
Others		1 (0.02)
Tumor size in MRI (mm)	44.27 (26.86)	
Histologic type		
ductal		60 (100)
Ki67 (%)	37.26 (18.83)	
Tumor grade		
G1		3 (5)
G2		19 (31.7)
G3		38 (63.3)
HR		
ER+		29 (48.3)
PR+		17 (28.3)
Number of suspicious lymph nodes by ultrasound		
1		23 (38.3)
2		12 (20)
3		4 (6.7)
4		6 (10)
> 4		15 (25)
cN		
N1		40 (66.7)
N2		17 (28.3)
N3		3 (5)
Radiologic response		
rCR-breast		35 (58.3)
rCR-axillary		46 (76.7)
Breast surgery		
Conservative surgery		20 (33.3)
Mastectomy		39 (65)
Axillary treatment		
SLNB		5 (8.3)
TAD		19 (31.7)
ALND		36 (60)
Pathologic response		
pCR-breast		23 (38.3)
pCR-axillary		51 (85)
Follow-up (months)	51 (13–108)	

*SD* standard deviation, *MRI* magnetic resonance imaging, *mm* millimeters, *HR* hormone receptors, *ER* estrogen receptor, *PR* progesterone receptor, *cN* clinical axillary nodes, *rRC* radiological complete response, *pCR* pathologic complete response, *SLNB* sentinel lymph node biopsy, *TAD* targeted axillary dissection, *ALND* axillary lymph node dissection

After NAC, 36 patients (60%) underwent ALND. Of these, 21 had initially presented with ≥ 4 suspicious lymph nodes, in accordance with clinical practice guidelines at that time. In the remaining 15 patients with 1–3 suspicious nodes at diagnosis, ALND was indicated in specific scenarios: 3 due to positive SLNB or TAD, 1 because of a suspicious level II lymph node (cN2), 3 due to additional suspicious nodes detected on positron emission tomography (PET)/CT, 1 because axillary drainage could not be achieved in lymphoscintigraphy, 1 patient with cutaneous involvement at diagnosis, and 1 case with lack of breast rCR in MRI or PET/computed tomography (CT).

**Fig. 2:**
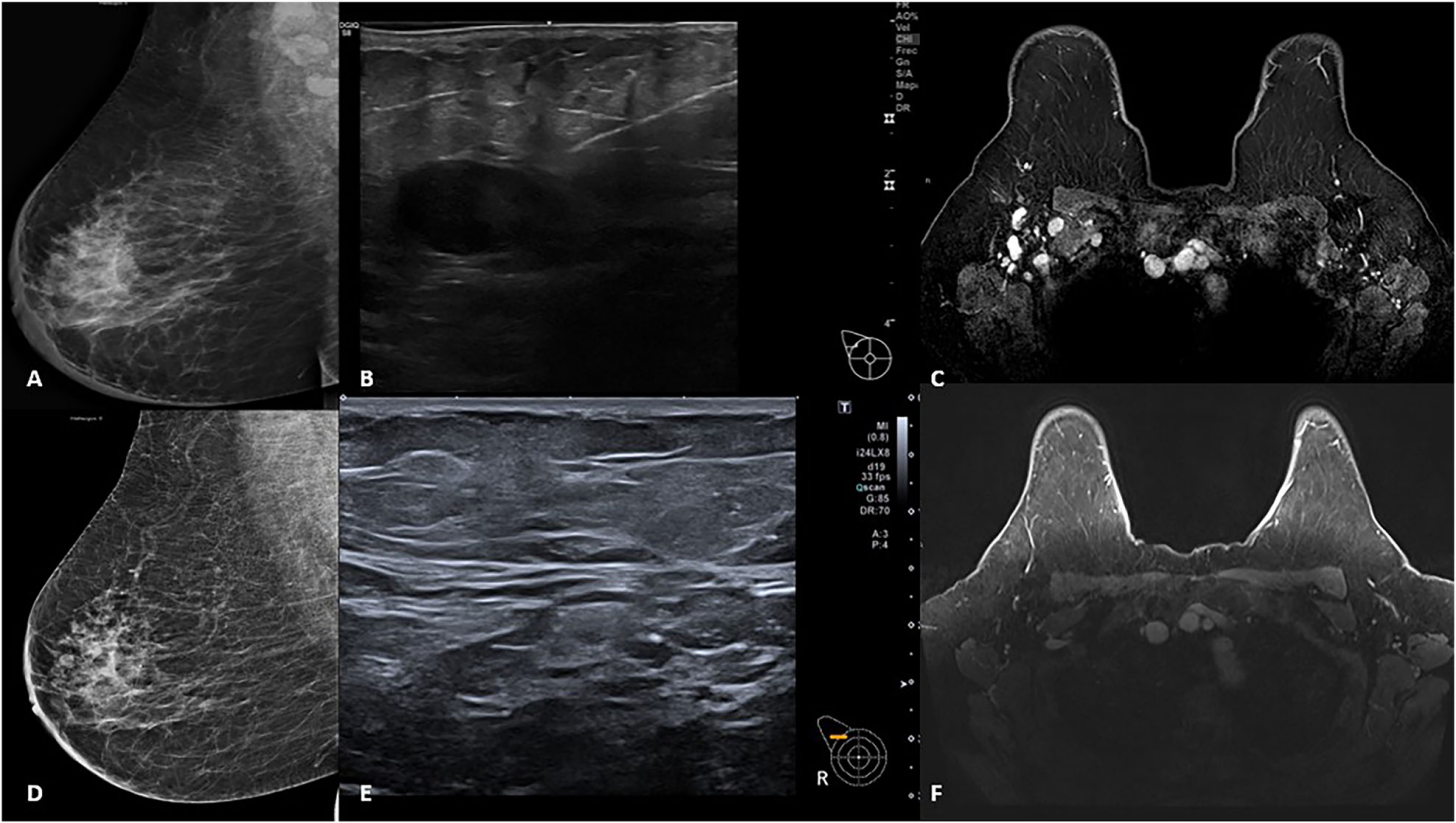
Axillary-rCR Clinical case. HER2+ breast cancer in a 68-year-old woman with invasive ductal carcinoma, grade 3, hormone receptor-negative, HER2+, and Ki-67 of 50%, presenting with more than four suspicious axillary lymph nodes at diagnosis (**A**–**C**), who achieved radiological and pCR after NAC (**D**–**F**). **A** Right MLO mammogram showing a retroareolar asymmetric density with enlarged axillary lymph nodes and generalized skin thickening. **B** Axillary ultrasound demonstrating a pathological lymph node with cortical thickening and fatty hilum replacement (Bedi type 6). **C** Axial contrast-enhanced T1-weighted fat-saturated MRI showing multiple pathological right axillary lymph nodes. **D** Follow-up mammogram (MLO view) showing disappearance of axillary lymphadenopathy and resolution of skin thickening. **E** Axillary ultrasound after NAC revealing lymph nodes with normal morphology, thin cortex, and preserved fatty hilum. **F** Axial contrast-enhanced T1-weighted fat-saturated MRI showing no residual pathological axillary lymph nodes

Following NAC, 51 patients (85%) achieved axillary pCR (ypN0), while nine patients (15%) had residual axillary disease (ypN + ). Among the patients who initially presented with 1–3 suspicious lymph nodes, 33/39 (84.6%) achieved axillary pCR. All patients with four suspicious nodes (6/6) achieved axillary pCR. Similarly, 12/15 (80%) patients with more than four suspicious nodes also achieved axillary pCR. Figure 2 presents an illustrative case of a patient with > 4 suspicious lymph nodes at baseline who achieved pCR following NAC.

## Univariate and multivariate analysis

The predictive factors for axillary pCR as assessed via the univariate and multivariate analyses are described in Table 2. The univariate analysis revealed that axillary rCR and breast rCR were significant predictors of axillary pCR (OR = 5.83; 95% CI: 1.30–26.12, *p* = 0.021 and OR = 6.79; 95% CI: 1.27–36.34; *p* = 0.025, respectively). Although the Ki-67 index also showed a trend toward association with axillary pCR (OR = 0.96; 95% CI: 0.90–1.01, *p* = 0.117), it did not reach statistical significance on the univariate analysis. Age, the number of affected lymph nodes, tumor grade, and hormone receptor status did not show significant associations with axillary pCR.

**Table 2:** Predictive factors for pCR-axillary

	Univariate analysis OR (95% CI)	*p*-value	Multivariable analysis OR (95% CI)	*p*-value
Age	1.04 (0.98–1.10)	0.237	1.09 (0.99–1.18)	0.070
Hormonal receptors	1.32 (0.29–6.02)	0.719		
Tumor grade (ref. grade 1)	0.00	0.999		
Tumor grade 2	1.00 (0.22–4.53)	1.000		
Tumor grade 3				
Ki67 (%)	0.96 (0.90–1.01)	0.117	0.90 (0.82–0.99)	0.045
rCR-axillary	5.83 (1.30–26.12)	0.021	17.98 (1.43–225.93)	0.025
Number of lymph nodes (ref. N° of lymph nodes 1)				
Number of lymph nodes 2	0.36 (0.03–4.04)	0.410	0.06 (0.02–1.74)	0.100
Number of lymph nodes 3	0.00	0.999	0.00	0.999
Number of lymph nodes 4	0.00	0.999	0.00	0.999
rCR-breast	6.79 (1.27–36.34)	0.025		
pCR-breast	10.38 (5.19–44.30)	0.998		

Univariate and multivariate analyses

*CI* confidence interval, *OR* odds ratio, *rCR* radiological complete response

To identify independent predictors of axillary pCR, a multivariable logistic regression analysis was performed that included the variables with clinical or statistical significance on the univariate analysis. Age and the number of lymph nodes were included in the multivariable model as confounding variables.

In the adjusted model, both axillary rCR and the Ki-67 index were identified as independent predictors of axillary pCR. The presence of axillary rCR was significantly associated with axillary pCR, as its absence increased the likelihood of not achieving axillary pCR by approximately 18-fold (OR = 17.98; 95% CI: 1.43–225.93, *p* = 0.025). This finding underscores the strong predictive capacity of radiological normalization of axillary nodes following NAC. Additionally, the Ki-67 index was directly correlated with a higher likelihood of achieving axillary pCR, indicating that tumors with greater proliferation showed a stronger pathological response (OR = 0.90; 95% CI: 0.82–0.99, *p* = 0.045) (Table 2).

## Model fit and validation

**Fig. 3:**
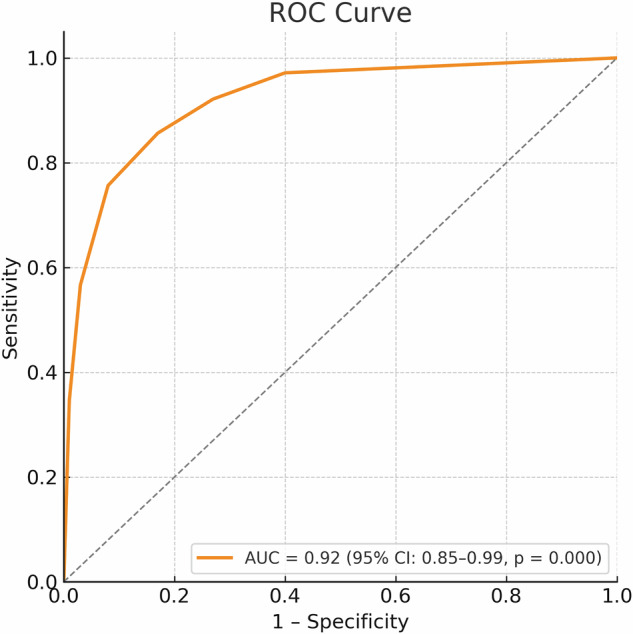
ROC curve. ROC curve evaluating the predictive performance of the model for axillary pCR. The curve plots sensitivity (*y*-axis) against 1-specificity (*x*-axis). The AUC is 0.92 (95% CI: 0.85–0.99; *p* = 0.000), demonstrating excellent discriminative ability

The multivariable model demonstrated good fit according to the Hosmer–Lemeshow test (*p* = 0.393), indicating adequate calibration. The ROC curve analysis yielded an AUC of 0.92 (95% CI: 0.85–0.99, *p* = 0.000), demonstrating the model’s excellent discriminative ability for predicting axillary pCR (Fig. 3).

## Performance metrics

Additional diagnostic performance metrics were calculated for axillary rCR and Ki-67. In the overall cohort, axillary rCR reached a sensitivity of 82.0% and a PPV of 91.1%, while specificity (55.6%) and NPV (35.7%) were lower. For Ki-67, using the cohort median (30%) as the cut-off, the total analysis showed a sensitivity of 82.0% and a PPV of 91.1%, with limited specificity (55.6%) and NPV (35.7%). Subgroup analyses revealed no relevant variations, either according to initial nodal burden (< 4 vs ≥ 4 suspicious nodes) or Ki-67 levels (< 30% vs ≥ 30%). Full results are presented in Table 3.

**Table 3:** Sensitivity, specificity, PPV, and NPV for axillary-rCR (overall and by nodal burden: < 4 vs ≥ 4 nodes) and Ki-67 (overall and by < 30% vs ≥ 30%)

	Sensitivity [IC 95%]	Specificity [IC 95%]	PPV [IC 95%]	NPV [IC 95%]
Axillary-rCR				
< 4 Suspicious nodes	81.8% [64.5–93] (27/33)	50% [11.8–88.2] (3/6)	90.0% [73.5–97.9] (27/30)	33.3% [7.5–70.1] (3/9)
≥ 4 Suspicious nodes	83.3% [58.6–96.4] (15/18)	66.7% [9.4–99.2] (2/3)	93.8% [69.8–99.8] (15/16)	40% [5.3–85.3] (2/5)
Total cohort	82.4% [69.1–91.6] (42/51)	55.6% [21.2–86.3] (5/9)	91.3% [79.2–97.6] (42/46)	35.7% [12.8–64.9] (5/14)
Ki67				
< 30	81.2% [54.4–96] (13/16)	40% [5.3–85.3] (2/5)	81.2% [54.4–96.0] (13/16)	40.0% [5.3–85.3] (2/5)
≥ 30	82.4% [65.5–93.2] (28/34)	75.0% [19.4–99.4] (3/4)	96.6% [82.2–99.9] (28/29)	33.3% [7.5–70.1] (3/9)
Total cohort	82.0% [68.6–91.4] (41/50)	55.6% [21.2–86.3] (5/9)	91.1% [78.8–97.5] (41/45)	35.7% [12.8–64.9] (5/14)

*PPV* positive predictive value, *NPV* negative predictive value

## Follow-up

The median follow-up was 51 months (range 13–108). Seven oncologic events were recorded: one patient developed axillary recurrence with distant metastases, one contralateral breast cancer, one ipsilateral breast recurrence without axillary involvement, two distant metastases, and two deaths. All the patients had been treated with mastectomy and ALND, except for the one with ipsilateral breast recurrence, who had undergone breast-conserving surgery with SLNB. Notably, no events were observed in patients treated with TAD.

## Discussion

This study observed a remarkable rate of axillary pCR (ypN0, post-treatment absence of nodal residual disease): it was achieved in 85% of patients. This is a testament to the efficacy of NAC combined with anti-HER2 agents in this specific breast cancer subtype. On the univariate analysis, axillary rCR showed a strong association with axillary pCR (OR = 5.83; 95% CI: 1.30–26.12, *p* = 0.021) while the Ki-67 index demonstrated a trend toward significance (OR = 0.96; 95% CI: 0.90–1.01, *p* = 0.117). The multivariable model confirmed axillary rCR and Ki-67 as independent predictive factors, with the absence of axillary rCR increasing the likelihood of residual axillary disease by approximately 18-fold (OR = 17.98; 95% CI: 1.43–225.93, *p* = 0.025). Additionally, the Ki-67 index was directly correlated with a higher probability of achieving axillary pCR (OR = 0.90; 95% CI: 0.82–0.99, *p* = 0.045), indicating that for each 1% increase in Ki-67, the odds of residual axillary disease decrease by approximately 10%. This suggests that tumors with a more aggressive biological behavior, indicated by a higher Ki-67, paradoxically demonstrate a greater sensitivity to neoadjuvant treatment. Age and the number of lymph nodes involved were identified as confounding factors, influencing the predictive strength of these variables. The model demonstrated strong discriminative ability with an AUC of 0.92 (95% CI: 0.85–0.99, *p* = 0.000), reinforcing its robustness in predicting axillary response.

These findings align with previous studies highlighting axillary rCR as a strong predictor of axillary pCR. Smith et al identified axillary rCR as a key factor associated with axillary pCR, reporting an OR of 6.21 (95% CI: 2.14–17.99, *p* < 0.001) [34]. Similarly, Johnson et al reported an OR of 5.47 (95% CI: 1.89–15.32, *p* < 0.01) for axillary rCR as a predictor of axillary pCR [35]. Furthermore, Samiei et al reported the effectiveness of noninvasive imaging in assessing axillary response after NAC, highlighting that axillary ultrasound had a sensitivity of 79% and a specificity of 88% in detecting pCR. In comparison, our results showed a slightly higher sensitivity (82%) but with a lower specificity (56%). Pislar et al specifically documented an OR of 4.89 (95% CI: 1.95–12.31) for the predictive value of ultrasound in detecting nodal response. In our series, axillary rCR achieved a PPV of 91%, further supporting its role as a reliable indicator of axillary pCR. This lends strength to the role of ultrasound in clinical decision-making [7, 25].

In terms of Ki-67, the results of this study corroborate those of Lee et al, Rais et al, and Fujita et al in showing a positive correlation between higher proliferation indices and better pathological response rates with an OR for Ki-67 as a predictor of pCR of 2.89 (95% CI: 1.32–6.32, *p* = 0.007), 3.12 (95% CI: 1.45–7.01, *p* = 0.004), and 3.74 (95% CI: 1.50–9.32, *p* = 0.005), respectively [9, 19, 36].

While these results demonstrate the potential of post-NAC axillary ultrasound to predict axillary pCR, a significant relationship was not found between the number of lymph nodes involved at diagnosis and the likelihood of achieving axillary pCR. These findings are in line with those of Yilmaz et al, who also reported no significant correlation between sentinel lymph node involvement and the number of positive axillary nodes. Together with the high percentage of patients that achieved axillary pCR after NAC, these findings suggest that the number of lymph nodes involved at diagnosis may not be a critical factor in determining the type of axillary surgery (ALND vs TAD) [21]. This is consistent with García-Tejedor et al’s findings, which explored the feasibility of omitting ALND in patients with an initially high nodal burden (cN2) who responded well to NAC [15]. Their results support the idea that in HER2+ and TNBC, in which high rates of axillary pCR are observed, surgical de-escalation strategies such as SLNB and TAD could be safe alternatives to ALND. Similarly, van Hemert et al evaluated the applicability of omitting ALND in patients with extensive nodal disease (≥ 4 involved nodes) who achieved pCR of the marking axillary lymph nodes with radioactive iodine seeds-marked lymph node following NAC. The findings demonstrated a low axillary recurrence rate (2.9%) and excellent oncologic outcomes (five-year overall survival: 95%), supporting de-escalation of axillary surgery in well-selected patients [37]. These findings provide further evidence for this study’s results that post-NAC axillary ultrasound might be a more relevant criterion for guiding surgical decision-making than the initial nodal burden at diagnosis.

Despite these promising findings, this study has limitations. As a single-center, retrospective study with a relatively small sample size, these results cannot be fully extrapolated to other populations. The high axillary pCR rate observed in our series (85%), slightly higher than the rates reported in previous studies, may limit the generalizability of our results. Additionally, the strict criteria for defining axillary rCR, namely complete normalization of lymph node morphology, may have led to underestimating the true pathological response rate. Recent consensus guidelines, such as those by Bernet et al and Burstein et al, suggest broader criteria for defining axillary rCR, including not only complete morphological normalization but also a significant reduction in lymph node size, cortical thinning, disappearance of an abnormal hilum, and absence of new suspicious features on imaging test, which might improve classification accuracy [13, 32]. Another limitation is the inherent variability of ultrasound-based assessments, as interobserver differences could affect the evaluation of nodal response.

Future research should focus on refining imaging criteria to improve the accuracy of axillary rCR detection and validating predictive models in larger multicenter cohorts. Prospective trials that evaluate the safety of omitting ALND in patients with favorable response profiles would also be valuable in order to confirm the long-term oncologic safety of this approach.

## Conclusion

In conclusion, this study’s findings identified axillary rCR in post-NAC ultrasound and Ki-67 as independent predictive factors of axillary pCR, underscoring their relevance in selecting patients who may benefit from less invasive surgical approaches. Given the high rate of axillary pCR observed, performing ALND based solely on the initial nodal burden may result in unnecessary morbidity without oncologic benefit. Instead, integrating axillary rCR as a criterion for selective axillary approaches, such as TAD, could optimize patient outcomes, avoiding unnecessary ALND in HER2+ patients who respond well to NAC.

## Abbreviations

ACOSOG: American College of Surgeons Oncology Group
ALND: Axillary lymph node dissection
AUC: Area under the curve
HER2+: Human epidermal growth factor receptor 2-positive
MLO: Mediolateral oblique (view)
NAC: Neoadjuvant chemotherapy
NPV: Negative predictive value
OR: Odds ratio
pCR: Pathological complete response
PET/CT: Positron emission tomography/computed tomography
PPV: Positive predictive value
rCR: Radiological complete response
ROC: Receiver operating characteristic
SD: Standard deviation
SLNB: Sentinel lymph node biopsy
TAD: Targeted axillary dissection
TNBC: Triple-negative breast cancer
